# Integrase-Mediated Recombination of the *veb1* Gene Cassette Encoding an Extended-Spectrum β-Lactamase

**DOI:** 10.1371/journal.pone.0051602

**Published:** 2012-12-10

**Authors:** Daniel Aubert, Thierry Naas, Patrice Nordmann

**Affiliations:** Service de Bactériologie-Virologie, INSERM U914 “Emerging Resistance to Antibiotics,” LabEx LERMIT, Hôpital de Bicêtre, Assistance Publique/Hôpitaux de Paris, Faculté de Médecine Université Paris-Sud, Paris, France; Saint Louis University, United States of America

## Abstract

The *veb1* gene cassette encodes the extended spectrum β-lactamase, VEB-1 that is increasingly isolated from worldwide Gram-negative rods. *Veb1* is commonly inserted into the variable region of different class 1 integrons in which it is always associated with a downstream-located *aadB* gene cassette encoding an aminoglycoside adenylyltransferase. In *Pseudomonas aeruginosa*, the majority of *veb1*-containing integrons also carry an insertion sequence, IS*1999* that is inserted upstream of the *veb1* gene cassette and disrupts the integron specific recombination site, *attI1*. Investigation of the recombination properties of the sites surrounding *veb1* revealed that insertion of IS*1999* reduces significantly the recombination frequency of *attI1* and that *veb1 attC* is not efficient for recombination in contrast to *aadB attC*. Subsequent sequence optimisation of *veb1 attC* by mutagenesis, into a more consensual *attC* site resembling *aadB attC*, successfully improved recombination efficiency. Overall, this work gives some insights into the organisation of *veb1-*containing integrons. We propose that IS*1999* and the nature of *veb1 attC* stabilize the *veb1* gene cassette environment likely by impairing recombination events upstream or downstream of *veb1*, respectively.

## Introduction

**Figure 1:**
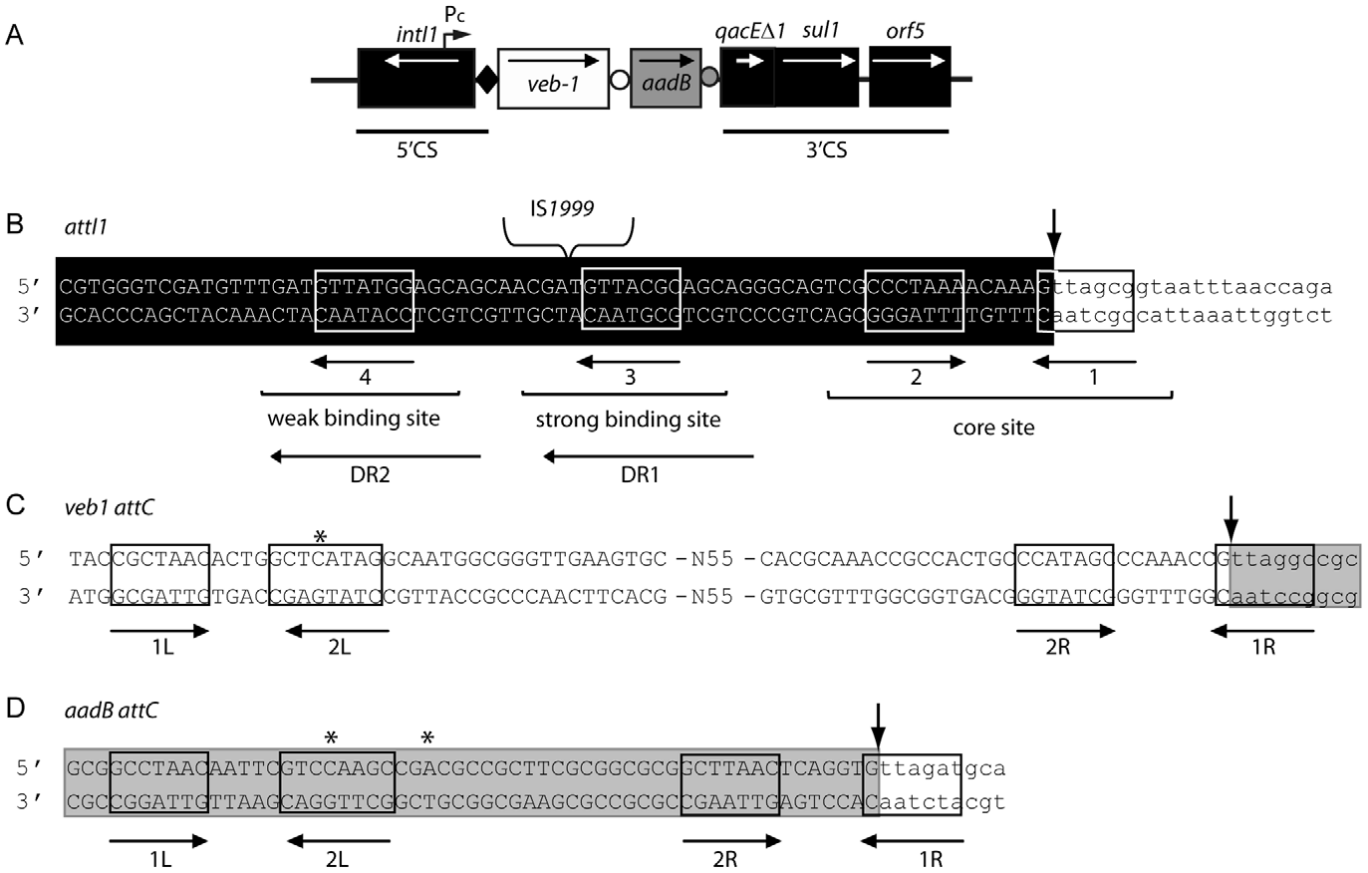
Schematic representations of the *veb1*-containing integron from *P. aeruginosa* 14 and of the recombination sites [**26**] **.** (A) *veb1*-containing integron from *P. aeruginosa* 14. The 5′ and the 3′-CSs are underlined. ORFs are shown as boxes with an arrow indicating the orientation of the coding sequence. The promoter P_c_ is indicated by a broken arrow. The black diamond represents *attI1* and circles represent *attC* sites. (B–D) Recombination sites. Sequences related to the 7-bp core site are boxed; their relative orientations are indicated with arrows. The crossover points are marked by vertical arrows. The region derived from the downstream-located cassette at the recombination point is in lower case. The extra-helical bases (EHBs) as defined by Bouvier *et al*. [18] are marked with an asterisk. (B) *attI1*: the nucleotides belonging to *attI1* are indicated in white on a black background. The experimentally determined strong and weak IntI1-binding sites and the pair of direct repeats, DR1 and DR2, as well as the location of the IS*1999* insertion in *attI1* are indicated. (C) *veb1 attC* and (D) *aadB attC*: 7-bp putative core sites (1L, 2L, 2R and 1R) related to the core site consensus as defined by Stokes et al. [14] are shown. The *aadB* gene cassette is boxed in grey.

Class 1 integrons are increasingly reported as a reservoir for antibiotic resistance genes in Gram-negative rods [1], [2]. These structures possess two conserved regions located on each side of a variable region consisting of integrated gene cassettes [1]–[3] (Figure 1A). The 5′ conserved segment (5′-CS) classically includes a gene encoding a site-specific recombinase of the DNA integrase family, *intI1*, the cassette integration site, *attI1*, and the promoter Pc, which is oriented toward the integration point of the gene cassettes and is responsible for gene cassette expression [2]–[6]. Class 1 integrons may not always contain the entire 3′ conserved segment (3′-CS), which typically includes along with an open reading frame of unknown function (*orf5*), the truncated disinfectant (*qacE*Δ*1*) and the sulfonamide (*sul1*) resistance determinants [1]–[3]. Gene cassettes from the variable region are composed of a gene, usually an antibiotic resistance gene, and a downstream recombination site known as *attC* site or 59-base element (59-be) [1]–[3]. Gene cassettes are independent units that are most often promoter-less. Consequently, gene expression levels depend on the cassette position in the integron. The further the cassette is relative to Pc, the lower its expression will be [4].

Gene cassettes are non-replicative mobile elements that exist under a free circular or integrated linear form [3], [7]. Site-specific recombination leading to gene cassette excision or integration is catalyzed by the integrase *IntI1*, which recognizes two structurally distinct sites, *attI1* and *attC* [7], [8]. The *attI1* site is particularly conserved and includes four integrase binding domains (Figure 1B). A pair of inversely oriented binding sites is located at the core site and two other integrase binding sites in direct repeat (DR1, DR2) are located further upstream [2], [9], [10]. A full *attI1* site containing four integrase binding domains is required for optimal recombination with an *attC* site [11], [12]. The *attC* sites that are associated with the gene cassettes are more complex and weakly related to each other [2]. They differ greatly in sequence and length but contain two pairs of inversely oriented integrase-binding domains (1L-2L and 2R-1R) [13], [14] (Figure 1C and 1D). Recombination mediated by IntI1 involves recognition of the bottom strand of the *attC* site [15]. Upon folding into a hairpin structure, single-stranded *attC* sites present an almost canonical core site consisting of 2L-2R and 1L-1R duplexes separated by a bulged area [2], [16]–[20]. However *attC* site recognition and proper interaction with the integrase are not dependent on canonical DNA but on the position of two extrahelical bases that interact with the integrase and originate from symmetrical folding of the bottom strand of *attC* [19].

Integrase-mediated recombinations between two *attC* sites, between two *attI1* sites, and between an *attI1* site and an *attC* site have been documented, the latter being the most efficient [8], [21]. During recombination the crossover point is located between the G and TT of the 7-bp core site motif, GTTRRRY, found at the 3′ end of the recombination sites [2], [14].

**Figure 2:**
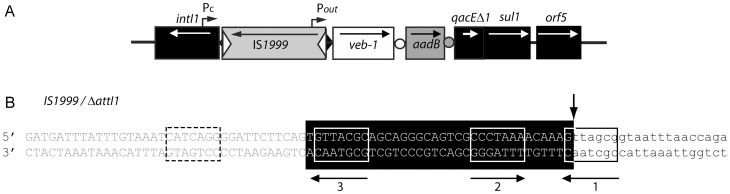
Schematic representation of the *veb1*-containing integron from *P. aeruginosa* 15 and of the disrupted *attI1* recombination site [**26**]. (A) *veb1*-containing integron from *P. aeruginosa* 15. ORFs are shown as boxes with an arrow indicating the orientation of the coding sequence. Promoters P_c_ and P_out_ are indicated by broken arrows. Disruption of *attI1* by IS*1999* is represented by a split black diamond; circles represent *attC* sites. The inverted repeats of IS*1999* are shown as empty triangles. (B) Sequence of the disrupted *attI1* site (Δ*attI1)*. The nucleotides belonging to IS*1999* and *attI1* are indicated in grey and in white on a black background, respectively. Sequences related to the 7-bp core site are boxed; their relative orientations are indicated with arrows. The crossover point is marked by vertical arrow. The region derived from the downstream-located cassette (*veb-1*) at the recombination point is in lower case. The 7-bp from IS*1999* replacing the fourth integrase binding site from *attI1* are shown in a dashed box.

Among the antibiotic resistance genes that are integron-located, the extended spectrum β-lactamase (ESBL) *bla*_VEB-1_ gene has been identified in a series of Gram-negative rods that are scattered worldwide [22]–[26]. At least seven different types of *veb1-*containing integrons were identified based on cassette content [22], [23], [26]. It is more than likely that the different *veb1*-containing integrons evolved from a common ancestor, however they have maintained interesting characteristics. In all cases, *veb1* is associated with a downstream-located *aadB* cassette encoding an aminoglycoside acetyltransferase (Figure 1A) [26]. An insertion sequence (IS*1999*) is inserted in the majority of the *veb1*-containing integrons characterized in *P. aeruginosa* [26], [27]. Upon insertion, IS*1999* disrupts the integron-specific recombination site, *attI1*, but provides an outward-directed promoter P_out_, which increases *bla*_VEB-1_ expression in *P. aeruginosa* (Figure 2A) [28]. Moreover, *veb1* has always been reported as the first cassette within the variable region of IS*1999*-containing integrons carrying different cassette arrays [26], [27].

The stability of the *veb1* environment was puzzling given the fact that the variable region of an integron is normally in constant evolution since it is subject to gene cassette rearrangement, loss and acquisition [3]. Cointegration assays were performed to determine the relative recombination efficiency of the different recombination sites present in the *veb1* vicinity (i.e. *attI1*, disrupted *attI1, veb1 attC* and *aadB attC*). This work revealed that *veb1 attC* and the disrupted *attI1* site of IS*1999*-containing integrons are not efficient for recombination and consequently might preserve the associations *veb1-aadB* and IS*1999*-*veb1* from being disrupted, respectively.

## Results

### The *veb1 attC* site is not efficient for recombination

Integrase-mediated recombination involving integrons located on multicopy plasmids can generate different recombination products including: (i) free circular DNA molecules comprising one or more gene cassettes, resulting from recombination between the gene cassette *attC* and either *attI1* or another *attC* site from another gene cassette [7]; (ii) cointegrates, resulting from recombination between two copies of the same plasmid. Different cointegrates can be formed depending on the sites that are available for recombination; (iii) gene duplications, which can arise by either insertion of a second gene copy encoded on a previously excised gene cassette or by formation and resolution of cointegrates (Figure S2). While the abundance of circular intermediates is very low, plasmid cointegrates and gene duplications are predominantly formed during recombination when intI1 is overexpressed [29]. Moreover, under these conditions gene cassettes that are unnecessary for bacterial growth are often excised and lost from the variable region [21].

**Figure 3:**
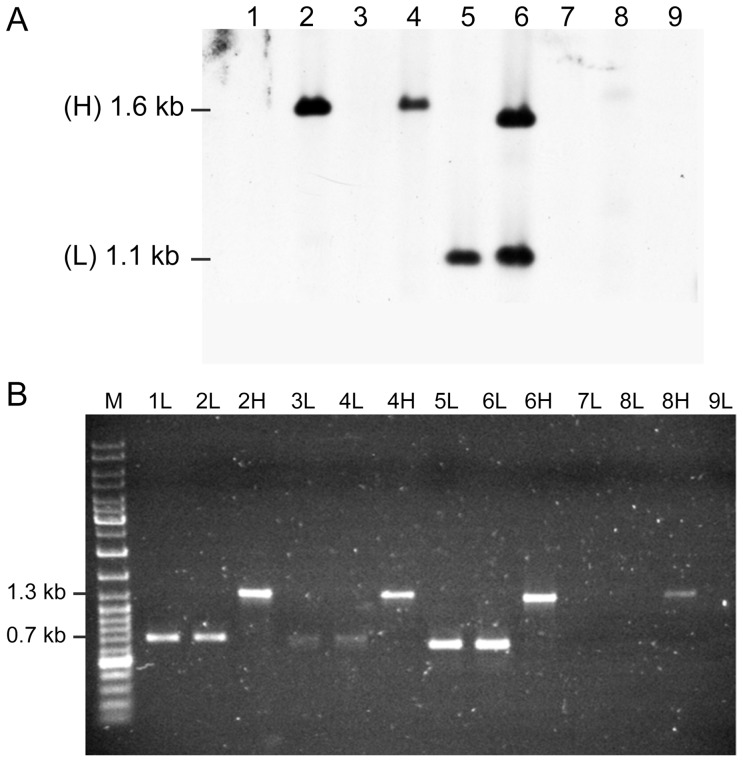
*Bla*_VEB-1_-specific hybridization and PCR amplifications of *veb1*-containing fragments. (A) *Bla*_VEB-1_-specific hybridization of small BspEI-digested DNA fragments from *E. coli* DH10B (p112.Kan) harboring pAttI.veb (1), pAttI.veb.aadB (2), pAttI.IS.veb (3), pAttI.IS.veb.aadB (4), pAttI.veb* (5), pAttI.veb*.aadB (6), pVeb (7), pVeb.aadB (8) and from *E. coli* DH10B (pTRC99A.Kan) harboring pAttI.veb.aadB (9) Locations of the low (L) 1.1-kb and high (H) 1.6-kb signals are indicated. (B) PCR amplifications of *veb1*-containing fragments using the outward directed primers VEBINV3-VEBINV2 and ca. 1.1-kb (L) and 1.6-kb (H) DNAs that were gel-extracted based on (A), as template.

The relative recombination efficiency of the sites present within different *veb1*-containing constructs was tested (Table 1 and Figure S1). Upon induction of integrase expression, circular DNA molecules (including plasmids, cointegrates and excised genes cassettes) were purified, digested with BspEI and separated according to their size on an agarose gel. The bottom part of the gel containing only small DNA molecules (<2-kb) was analysed and *veb1*-containing products were detected by hybridization using a *bla*_VEB-1_ specific probe (Figure 3A). Our experimental conditions specifically allow for detection of recombination events that involved an *attI1* and an *attC* site but do not distinguish whether the product is an excised cassette, a gene cassette duplication or a cointegrate (Figure S2).

**Table 1:** Strains and plasmids.

Strain or plasmid	Relevant characteristics^a^	Source and/or reference
Strains		
*P. aeruginosa*		
Strain # 14	Clinical isolate containing class 1 integron, *veb1*	[26]
Strain # 15	Clinical isolate containing class 1 integron, IS*1999*, *veb1*, *aadB*	[26]
*E. coli*		
DH10B	F– *mcr*A Δ(*mrr*-*hsd*RMS-*mcr*BC) Φ80*lac*ZΔM15 Δ*lac*X74 *rec*A1 *end*A1 *ara*D139 Δ(*ara leu*) 7697 *gal*U *gal*K *rps*L *nup*G λ–	Life Technologies
DH10B-Rif	DH10B, Rif^R^	Laboratory stock
Plasmids		
R388	33kb IncW plasmid containing class 1 integron In3 (*dfrB2, orfA*), Tra+, Tmp^R^	[38]
p112	pTRC99A:: *intI1*, expression vector for integrase, P*trc*, Amp^R^	[33]
p112.Kan	p112 derivative, expression vector for integrase, P*trc, intI1*, Kan^R^	This study
pTRC99A.Kan	p112.Kan derivative without *intI1*, Kan^R^	This study
pBBR1MCS.3	Broad host range cloning vector, Tet^R^	[34]
pVeb	class 1 integron containing *veb1* in pBBR1MCS.3, Caz^R^, Tet^R^	This study
pVeb.aadB	class 1 integron containing *veb1, aadB* in pBBR1MCS.3, Caz^R^, Tet^R^	This study
pAttI.veb	class 1 integron containing, *attI1, veb1* in pBBR1MCS.3, Caz^R^, Tet^R^	This study
pAttI.veb.aadB	class 1 integron containing *attI1, veb1, aadB* in pBBR1MCS.3, Caz^R^, Tet^R^	This study
pAttI.IS.veb	class 1 integron containing disrupted *attI1*, IS*1999, veb1* in pBBR1MCS.3, Caz^R^, Tet^R^	This study
pAttI.IS.veb.aadB	class 1 integron containing disrupted *attI1*, IS*1999, veb1, aadB* in pBBR1MCS.3, Caz^R^, Tet^R^	This study
pAttI.veb*	pAttI.veb derivative, sequence modification of *veb1 attC* to match *aadB attC* (veb1 attC*)	This study
pAttI.veb*.aadB	pAttI.veb.aadB derivative, sequence modification of *veb1 attC* to match *aadB attC* (veb1 attC*)	This study
pAttI.veb^Δ^	pAttI.veb derivative, *veb1* with shorter *veb1 attC (veb1 attC* ^Δ^)	This study
pAttI.veb'	pAttI.veb derivative, sequence modification of the integrase binding sites 2L and 2R from *veb1 attC (veb1 attC*')	This study

a Antibiotic resistance: Amp^R^, ampicillin; Caz^R^, ceftazidime; Kan^R^, kanamycin; Rif^R^, rifampin; Tet^R^, tetracycline; Tmp^R^, trimethoprim.

Plasmid pAttI.veb contains a full *attI1* site, the *veb1* cassette and a truncated *aadB* cassette, while pAttI.veb.aadB contains, in addition to *attI1* and *veb1*, a full-length *aadB* cassette. While *attI1* and *veb1 attC* sites are theoretically the only sites available for recombination in pAttI.veb, pAttI.veb.aadB offers an additional recombination site, *aadB attC*. Recombination between *attI1* and *veb1 attC* leading to either *veb1* cassette excision, duplication or plasmid cointegration should produce, after BspE1 digestion, a *veb1*-containing DNA fragment of 1.1-kb. (Figure S2 and S3). However, no such product was observed with pAttI.veb and pAttI.veb.aadB, suggesting that *veb1 attC* is not efficient for recombination (Figure 3A lanes 1 and 2). Instead, a 1.6-kb product was clearly detected using circular DNA extracts from *E. coli* (pAttI.veb.aadB) (Figure 3A lane 2). Additional experiments confirmed that the 1.6-kb product was integrase-mediated since it was only detected when IntI1 was expressed (Figure 3A lanes 2 and 9).

A more sensitive approach using PCR amplification was used. Circular DNA molecules were purified, digested and separated on an agarose gel as before but agarose gel slices were cut at the expected migration of the 1.1-kb and 1.6-kb DNA fragments. Gel extracted DNAs were then subjected to PCR using outward-directed *bla*_VEB-1_ specific primers. These primers are located on each side of the BspE1 restriction site and specifically allow for amplification of *veb1*-containing DNA fragments that were linearized (excised gene cassettes) or released upon BspE1 restriction from cointegrates and plasmids with *veb1* duplication (Figure S2 and S3).

**Figure 4:**
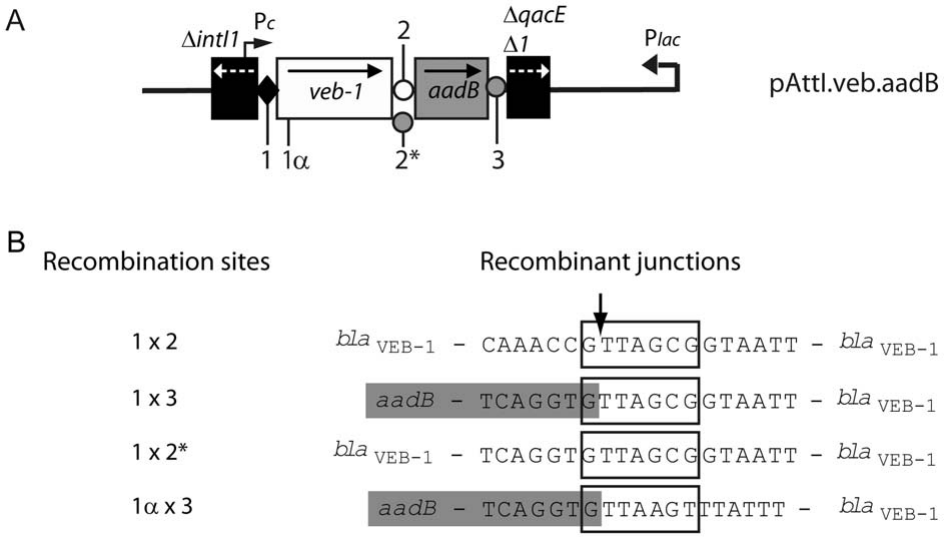
Recombinant junction analysis. (A) Representation of the pAttI.veb.aadB recombinant plasmid. The location of the different crossover points involved in recombination are indicated by a number: (1) 7-bp core site of *attI1-veb1*; (1α) secondary recombination site found within the *bla*_VEB-1_ coding sequence; (2), (2*) and (3) 7-bp site found at the veb1 attC-aadB, veb1 attC*-aadB and *aadB attC-qacEΔ1* junctions, respectively. (B) Sequencing results of the recombinant junctions from experimentally isolated *veb1*-containing recombination products. The numbers shown on the left indicate the recombination sites that were involved to create the recombinant junctions. Sequences related to the 7-bp core site are boxed. The crossover point is marked by vertical arrow. The nucleotides belonging to the *aadB* gene cassette are boxed in grey.

PCR amplification using DNA extracted from the 1.1-kb gel slices from *E. coli* (pAttI.veb) and *E. coli* (pAttI.veb.aadB) yielded a 0.7-kb PCR product (Figure 3B lanes 1L and 2L). Sequencing indicated that it corresponded to *veb1* and further analysis of the recombinant junction confirmed that recombination occurred precisely between *attI1* and *veb1 attC* (Figure 1B, 1C and 4). Similarly, PCR amplifications using the 1.6-kb product from *E. coli* (pAttI.veb.aadB) yielded a 1.3-kb fragment (Figure 3B lane 2H). Sequencing revealed that it contained both *veb1* and *aadB* gene cassettes and that recombination occurred precisely between *attI1* and *aadB attC* (Figure 1B, 1D and 4). The predominant formation of the 1.6-kb product also indicated that *aadB* remained mostly adjacent to *veb1* after recombination.

These results underline the weak activity of *veb1 attC* for recombination and the stability of the association *veb1-aadB.* Both *veb1 attC* and *aadB attC* can recombine with *attI1.* However in contrast to *veb1 attC, aadB attC* is highly efficient for recombination as it is favored over *veb1 attC* during integrase-mediated recombination.

Plasmids pVeb and pVeb.aadB containing a truncated *attI1* site with only the 7-bp core site motif (GTTAGCG) at the junction with the *veb1* gene cassette were tested for recombination (Figure 1B and S1). Surprisingly, recombination products were detected in circular DNA extracts from *E. coli* (pVeb.aadB) albeit at low levels (Figure 3A lane 8 and 3B lane 8H). Sequencing revealed that recombination occurred between *aadB attC* and a secondary site (1α) instead of the expected GTTAGCG motif of the *veb1* gene cassette (Figure 4). This secondary recombination site (GTTAAGT) is homologous to a consensus core site GTTRRRY and is located 32-bp downstream of the translation initiation codon of *bla*_VEB-1_. Thus, recombination resulted in a truncated *veb1* gene cassette associated to *aadB* (Figure 4). Despite several attempts, we were not able to detect by PCR any product containing the truncated *veb1* cassette alone (Figure 3B lanes 7L and 8L). As expected, the 7-bp core site is not sufficient to support precise recombination with *attC*.

### Sequence optimisation of *veb1 attC* improves recombination

**Figure 5:**
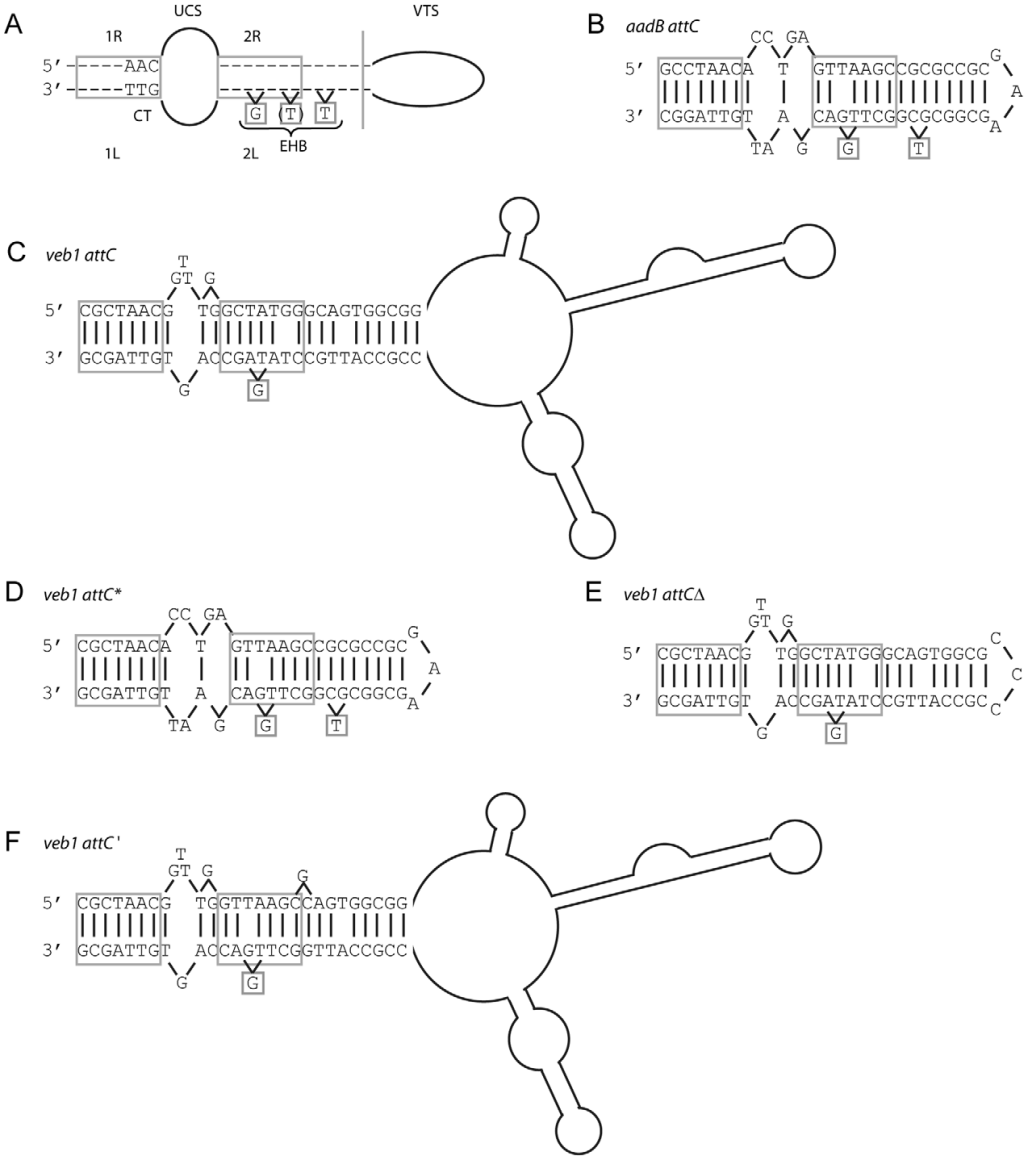
Bottom strand secondary structures. (A) consensus *attC* structure as of Cambray et al. [2]; (B) *aadB attC*; (C) *veb1 attC*; (D) veb1 attC*; (E) *veb1 attC^Δ^*; and (F) *veb1 attC*'. The putative IntI1 binding domains are marked with boxes and the inverted repeats 1L-1R and 2L-2R are indicated. The protruding G that determines the recombination strand present in 2L and the protruding T that increases the recombination efficiency, are also boxed. CT, conserved triplet; UCS, unpaired central spacer; EHB, extrahelical base; VTS, variable terminal structure. Folded representations were based on secondary structures obtained thanks to the mfold Web Server. The folded structure of the veb1 attC* site was similar to the folded structure of *aadB attC* and possessed a free energy of −30.3 Kcal/mol.

The *aadB attC* site fits closely to the consensus sequence of an *attC* site [2], [14]. It is 60-bp long and made of two nearly perfect inverted repeats, which are bounded by sequences matching precisely the consensus RYYYAAC/GTTRRRY (Figure 1D). Moreover, the bottom strand of *aadB attC* contains the two extrahelical bases (T-N_6_-G) found in the most easily excisable *attC* sites [30] (Figure 5). Therefore it is not surprising that *aadB attC* worked efficiently in our recombination assays. Folding of the bottom strand of *veb1 attC* revealed a characteristic secondary structure with the three structural elements common to *attC* sites [2] (Figure 5). However, *veb1 attC* presents two striking differences as compared to *aadB attC*: (i) the *veb1 attC* site (133-bp) has a longer variable terminal structure (VTS) and (ii) the two putative integrase binding sites 2L and 2R diverge from the *attC* site consensus sequence (Figure 1C).

The DNA segment from *veb1 attC* that is located between the 1L and 1R binding sites was replaced with the one from *aadB attC*, thus reducing the size and restoring the 2L and 2R consensus binding sites in *veb1 attC* (veb1 attC*) (Figure 5D). In this configuration, ca. 1.1-kb products were strongly detected with pAttI.veb* or pAttI.veb*.aadB (Figure 3A lane 5 and 6), which indicate that recombination between *attI1* and veb1 attC* occurred. Moreover, in cells harboring pAttI.veb*.aadB, *veb1-* and *veb1-aadB-*containing recombination products were detected at similar levels (Figure 3A lane 6) suggesting that veb attC* and *aadB attC* have a similar recombination efficiency. Sequencing confirmed that precise recombination had taken place and involved either the veb1 attC* site or the *aadB attC* site (Figure 4).

These results indicate that the sequence located between the 1L and 1R sites from *veb attC* is not optimal for recombination. The *veb1 attC* site was further modified to determine whether the long intermediate region located between the 2L and 2R integrase binding sites or the 2L and 2R sequences that diverged from an *attC* consensus sequence was responsible for the low recombination efficiency of *veb attC*. Two different *veb1 attC* sites named *veb1 attC* ^Δ^ and *veb1 attC*' were generated. The DNA segment located between the 2L and 2R binding sites from *veb1 attC* was reduced to 20-bp as found in *aadB attC* giving rise to *veb1 attC* ^Δ^ (Figure 5E). In *veb1 attC*', the 2L and 2R sequences were modified to match the 2L and 2R sequences found in *aadB attC* (Figure 5F).

**Figure 6:**
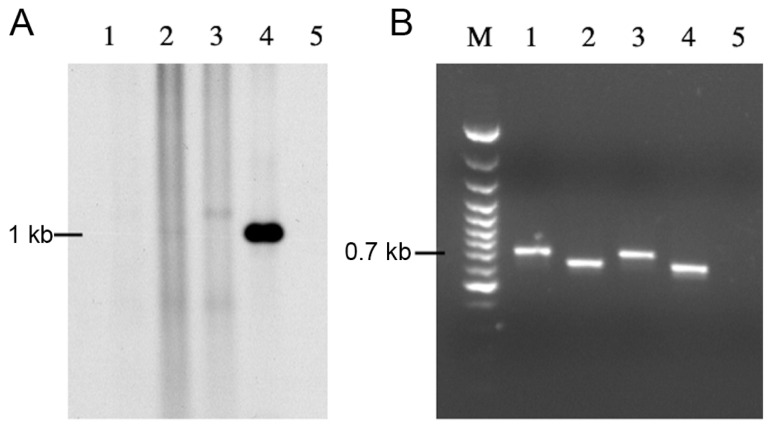
*veb1*-containing recombination products depending on *veb1 attC* variants. of *veb1*-containing fragments. (A) *Bla*_VEB-1_-specific hybridization of small BspEI-digested DNA fragments from *E. coli* DH10B (p112.Kan) harboring pAttI.veb' (1), pAttI1.veb^Δ^ (2), pAttI.veb (3), pAttI.veb* (4), and from *E. coli* DH10B (pTRC99A.Kan) harboring pAttI.veb (5). (B) PCR amplifications of *veb1*-containing fragments using the outward directed primers VEBINV3-VEBINV2 primers and the ca. 1.1 kb DNAs that were gel extracted from (A) as template.

Plasmids pAttI.veb^Δ^ and pAttI.veb' were tested for recombination (Figure 6). In contrast to veb1 attC*, veb1 attC ^Δ^ and *veb1 attC*' did not allow for the detection of *veb1*-containing recombination products by hybridization (Figure 6A lanes 1–2 and 4). However, *veb1*-containing recombination products were detected after PCR amplification indicating that *veb1 attC* ^Δ^ and *veb1 attC*' were functional (Figure 6B lanes 1–2). Overall, *veb1 attC* ^Δ^, *veb1 attC*' and wild type *veb1 attC* had similar activities indicating that modifications made to reduce the VTS or change the 2L and 2R sequences did not improve the recombination efficiency.

### IS*1999* insertion decreases *attI1* recombination efficiency

In *P. aeruginosa*, the *attI1* site of many *veb1*-containing integrons is disrupted by IS*1999* in such a way that only the last 34-bp of the site (containing three integrase binding sites) remain adjacent to *veb1* (Figure 2B). Using circular DNA isolated from *E. coli* (pAttI.IS.veb.aadB), *veb1*-containing recombination products that resulted from recombination between the disrupted *attI1* and *aadB attC* were detected by hybridization (Figure 3A lane 4). Recombination products involving the disrupted *attI1* and *veb1 attC* were only detected after PCR amplification (Figure 3B lanes 3L and 4L).

These results suggest that the *attI1* site disrupted by IS*1999* insertion is still functional for recombination. Nevertheless, based on signal intensity, the recombination efficiency of the disrupted *attI1* site seemed significantly lowered in comparison to a full-length *attI1* site (Figure 3A lanes 2 and 4, 3B lanes 1L and 3L).

### Recombination frequencies

Activities of the sites present within different *veb1*-containing constructs (Table 1 and Figure S1) were assayed in vivo by measuring the frequency of recovery of cointegrates formed between the test plasmids and plasmid R388. The self-conjugative plasmid R388 (TmpR, Tra+) includes an integron (In3) that contains the *dfrB2* cassette conferring resistance to trimethoprim (Tmp) followed by an open reading frame, *orfA*, of unknown function.

*Veb1*-containing constructs (conferring resistance to ceftazidime, Caz) were introduced into a rifampin sensitive *E. coli* containing the integrase expressing plasmid and plasmid R388. Upon induction of integrase expression, cointegrates resulting from recombination between the plasmid-located *veb-1* integrons and the recipient integron In3 located on plasmid R388 were predominantly formed. Plasmid R388 and cointegrates were transferred by conjugation into *E. coli* DH10B-Rif (rifampin resistant) and cointegration frequencies were measured as ratios of cointegrates (CazR-RifR) to total R388 transconjugants (TmpR-RifR) (Table 2).

**Table 2:** Cointegration and cassette integration frequencies.

Plasmid^a^	Plasmid cointegration frequency^b^	Cassette integration frequency	Number of CazR-RifR- TetS colonies^e^	*veb1* gene cassette insertion	*veb1–aadB* gene cassette insertion
pAttI.veb.aadB + pTRC99A.Kan	0	-	-	-	-
pBBR1MCS.3 + p112.Kan	0	-	-	-	-
pVeb + p112.Kan	4.41×10^−5^ (+/−1.96×10^−5^) c	ND^d^	0	-	-
pVeb.aadB + p112.Kan	2.15×10^−3^ (+/−1.80×10^−4^)	ND	0	-	-
pAttI.Veb + p112.Kan	7.35×10^−4^ (+/−3.26×10^−4^)	ND	0	-	-
pAttI.veb.aadB + p112.Kan	3.31×10^−3^ (+/− 1.89×10^−4^)	1.44×10^−4^	21	1	20
pAttI.IS.veb + p112.Kan	1.23×10^−4^ (+/−3.36×10^−6^)	ND	0	-	-
pAttI.IS.veb.aadB + p112.Kan	3.79×10^−3^ (+/−.10×10^−4^)	ND	0	-	-
pAttI.veb* + p112.Kan	1.37×10^−2^ (+/− 2.52×10^−3^)	1.70×10^−4^	6	6	-
pAttI.veb*.aadB + p112.Kan	6.25×10^−3^ (+/− 7.53×10^−4^)	2.07×10^−4^ (1.55×10^−4^)^f^	16	12	4

a Donor strains also contain R388; pTRC99A.Kan did not expressed IntI1 (negative control); p112.Kan (a pTRC99A.Kan derivative) expressed IntI1. The pVeb, pVeb.aadB, pAttI.veb, pAttI.veb.aadB, pAttI.IS.veb, pAttI.IS.veb.aadB, pAttI.veb* and pAttI.veb*.aadB were used for recombination (Figure S1). pBBR1MCS.3, cloning vector (negative control).

b The limit of detection of our assay is <1.5×10^−5^.

c Standard deviations calculated from three independent experiments.

d ND, Not determinable.

e up to 484 CazR, RifR transconjugants were tested for tetracycline sensitivity.

f Integration frequency of the *veb1* gene cassette alone.

Plasmid cointegration was IntI1 mediated since CazR-RifR cointegrates were only recovered from strains over-expressing the integrase. In plasmid pVeb, *veb1 attC* is the only site available for recombination with either *attI1* or an *attC* site from In3. Cointegrates (pVeb::R388) were selected at a very low frequency (4.41×10^−5^) close to the limit of detection of our assay, thus reflecting the inefficiency of *veb1 attC* for recombination. As compared to pVeb, the presence of *aadB attC* in pVeb.aadB, led to a 50-fold increase in the cointegration frequency, confirming that *aadB attC* is more efficient for recombination than *veb1 attC*. Comparison between (pVeb::R388) and (pAttI.IS.veb::R388) revealed that the disrupted *attI1* site is 3-times more efficient for recombination than the 7-bp core site from pVeb. However, insertion of IS*1999* into *attI1* (pAttI.IS.veb) led to a 6-fold decrease in the cointegration frequency, as compared to plasmid pAttI.veb. Sequence optimisation of *veb1 attC* into veb1 attC* led to an almost 20-fold increase in the cointegration frequency of pAttI.veb* as compared to pAttI.veb. Similar cointegration frequencies were obtained with plasmids pVeb.aadB, pAttI.veb.aadB, pAttI.IS.veb.aadB and pAttI.veb*.aadB. All these constructs contain the highly efficient recombination site *aadB attC* that was likely mainly involved in cointegration.

For each plasmid tested, several cointegrates were analyzed by PCR to determine which sites (i.e. attI1, veb1 attC, veb1 attC* or *aadB attC* from the *veb1*-containing plasmids) were involved in the recombination. As expected, all the pVeb::R388 and pVeb.aadB::R388 cointegrates involved *veb1 attC* and *aadB attC*, respectively. Cointegrates pAttI.veb::R388 and pAttI.IS.veb::R388 were formed by recombination using *attI1* or *veb1 attC*. All the pAttI.veb.aadB::R388 and pAttI.IS.veb.aadB::R388 cointegrates involved *aadB attC* and retained the *aadB* cassette in association with *veb1*. All the pAttI.veb::R388 cointegrates involved veb1 attC* and the pAttI.veb.aadB::R388 cointegrates had systematically lost the *aadB* cassette. Since *aadB* cassette excision could occur before or after cointegration, we could not determine whether pAttI.veb.aadB::R388 arose by cointegration involving veb1 attC* or *aadB attC*_._

CazR-RifR transconjugants were further screened for tetracycline susceptibility to discriminate cointegrates from precise *veb1* insertion into In3. In contrast to cointegration, which results from one recombination event; cassette insertion results from two recombination events (cassette excision-integration or plasmid cointegration-resolution) and occurs at a lower frequency. Despite most colonies (>95%) were resistant to tetracycline, few CazR-RifR-TetS transconjugants were identified. CazR-RifR-TetS transconjugants were recovered only from donor cells containing pAttI.veb.aadB, pAttI.veb* and pAttI.veb*.aadB plasmids with similar frequencies (Table 2). Each of these constructs has the particularity to carry at least two efficient sites that were used during cassette insertion. More than 95% of the CazR-RifR-TetS transconjugants recovered from cells containing pAttI.veb.aadB had integrated *veb1* along with *aadB*, demonstrating that *veb1* is co-mobilized with *aadB*. Using plasmid pAttI.veb*.aadB, both *veb1* and *veb1-aadB* insertions were found, however *veb1* inserts were mostly recovered indicating that *veb1* recombines efficiently and independently of *aadB* when it contains veb1 attC*.

## Discussion

Recombination activities of the sites surrounding the *veb1* gene cassette were investigated by using two independent recombination assays. It is noteworthy that experiments were performed in *E. coli* DH10B, which contains an inactivated form of RecA (RecA1) excluding recombination between homologous sequences. Thus, despite plasmids share some sequence identity, cointegration is unlikely to have occurred by homologous recombination but is truly the result of site-specific recombination mediated by the integrase.

Dissemination and acquisition of a single gene cassette within the variable region of integrons is the result of integrase-mediated recombination between two different sites, one of which is the gene cassette *attC* [7]. We demonstrate here that *veb1 attC* found within the *veb1* gene cassette, is not efficient for recombination implying that *veb1* alone is not highly mobilizable. Our cointegration experiments also revealed that *aadB attC* is favored over *veb1 attC* as it is almost exclusively involved in recombination when both gene cassettes are present on a plasmid. Despite no selection, *aadB* remained associated to wild type *veb1* in all the cointegrates tested, while gene cassettes that are unnecessary for bacterial growth, are often unstable and lost from the variable region when IntI1 is overexpressed [21]. Gene cassettes are often considered as independent units but their excision depends greatly on the recombination sites that are flanking them. Therefore, it was anticipated that *aadB* would be rarely excised since *aadB* excision require the involvement of *veb1 attC*. Stability of the association *veb1-aadB* within integrons is likely preserved by the nature of *veb1 attC*, which impairs not only *aadB* excision but also new insertions of gene cassettes in between them. Our cassette integration assay in plasmid R388 showed that the association *veb1-aadB* is to the benefit of *veb1*, which is mobilized along with *aadB* thanks to the high recombination efficiency of *aadB attC*.

The *aadB attC* site is 50 times more efficient for recombination than *veb1 attC*. Sequence optimisation of *veb1 attC* into a more consensual *attC* site resembling *aadB attC* (veb1 attC*) improved drastically the recombination efficiency and allowed independent excision of *aadB* from *veb1*-containing integrons. This experiment revealed also that the features responsible for the weak activity of *veb1 attC* reside within the DNA sequence located between the 1L and 1R integrase binding sites. Two major differences between *veb1 attC* and *aadB attC* were found in this region and concerned: (i) the 2L and 2R integrase binding sites, which sequences in *veb1 attC* diverged from the consensus and (ii) the length of the variable terminal structure (VTS). However, modification of one or the other in *veb1 attC* was not sufficient to improve recombination activity. While VTS seem to have a minor role in the *attC* recombination efficiency, other structural features including the unpaired central spacer (UCS) bulge shape and two extrahelical bases (EHBs) appear to be important for recognition and recombination rate [18]. In contrast to *aadB attC, veb1 attC* presents slightly larger UCS. Also, while the EHBs in *aadB attC* are 6 nucleotides apart (T-N_6_-G) as found in the *attC* sites flanking cassettes that are efficiently excised by IntI1 [30], *veb1 attC* contains only one EHB (G). It has been recently demonstrated that the identity and spacing of the EHBs in the *attC* sites has a pronounced effect on the efficiency of cassette excision [30]. The relevance of these structural differences in the activity of *veb1 attC* remains to be established. It is also possible that the recombination efficiency of *veb1 attC* is not affected by only one of these features but by several of them.

Several studies have shown that a full *attI1* site containing the four integrase binding sites was required for high efficiency recombination with *attC* and that progressive 5′ deletions reaching closer to the 7-bp core site of *attI1* leads to decreased recombination rates [11], [12], [31]. Insertion of IS*1999* into *attI1* displaces only the weak integrase binding site located in DR2. Accordingly, the disrupted *attI1* is still sufficient to support recombination albeit at a lower frequency than a full *attI1* site. Analysis of the bases from IS*1999* replacing part of *attI1* did not reveal any obvious 7-bp core site sequence, which could have compensated for the loss of the fourth integrase binding site. Also, by reducing the recombination rate of *attI1,* IS*1999* likely increases the stability of *veb1* and consequently *aadB* at the first positions within the variable region by impairing integration of new gene cassettes upstream of *veb1* and excision of *veb1-aadB.* However, since the disrupted *attI1* still supports recombination, new gene cassettes could be inserted at the first position and benefit from the additional promoter P_out_ located in IS*1999* for their expression [28].

Several studies have demonstrated that integrase is also able to catalyse recombination between one specific site (*attI1* or *attC*) and non-specific secondary sites conforming to the consensus GNT at a very low frequency [12], [21], [31]. Moreover, limitation of *attI1* to the 7-bp core site is insufficient for determining recombination specificity alone and leads to the formation of cointegrates owing to recombination with secondary sites [12]. Using plasmid pVeb.aadB, we demonstrated upon PCR amplification that *aadB attC* is able to recombine with the secondary site (GTTAAGT) located within the *bla*_VEB-1_ gene. This event resulting in a truncated *veb1* gene cassette is extremely rare since it has been detected only once in our repeats. The region that is truncated in *bla*_VEB-1_ encodes the first eleven amino acids of the pre-enzyme VEB-1 and includes the translation initiation codon and most of the signal peptide. Even upon insertion in an environment providing transcription and translation signals, it is unlikely that the protein encoded by the truncated *veb1* cassette would be functional in vivo since it would not be properly targeted to the periplasm.

Overall, this work gives some insights into the organisation of *veb1-*containing integrons that are widespread among Gram-negative bacteria. It is more than likely that the different *veb1*-containing integrons evolved from a common ancestor presenting an early association *veb1-aadB*. It is also possible that *aadB* is at the origin of the *veb1* gene cassette recruitment and of the co-mobilization of *veb1-aadB* into class 1 integrons. Even though *veb1*-containing integrons are still subject to gene cassette rearrangements, we propose that IS*1999* and the nature of *veb1 attC* stabilize the *veb1* gene cassette environment likely by impairing recombination events upstream or downstream of *veb1*, respectively.

## Materials and Methods

### Bacterial strains, plasmids and culture conditions

Bacterial strains and plasmids used in this study are listed in Table 1. The clinical strains *P. aeruginosa* 14 and 15 carrying different *veb1*-containing class 1 integrons were from the Siriraj Hospital, Bangkok, Thailand [26]. The recombination deficient strain *E. coli* DH10B (Life Technologies, Eragny, France) was used as bacterial host in electroporation experiments. The conjugative plasmid R388 (TmpR, Tra+) includes an integron (In3) that contains the *dfrB2* cassette conferring resistance to trimethoprim (Tmp) followed by an open reading frame, *orfA*, of unknown function [32]. Plasmid p112 (a pTRC99A derivative) was a gift from D. Mazel [33]. This plasmid contains the *intI1* gene under the control of an IPTG-inducible synthetic P_trc_ promoter. *E. coli* DH10B harboring various plasmids and *E. coli* DH10B-Rif (rifampin resistant) were used for conjugation experiments. The low-copy number cloning vector, pBBR1MCS.3 was used for cloning experiments [34]. Bacterial cells were grown in Trypticase Soy (TS) broth or onto TS agar plates (Sanofi Diagnostics Pasteur, Marnes-La-Coquette, France) with antibiotics when needed.

### Antimicrobial agents

The antimicrobial agents and their sources were as follows: ceftazidime, GlaxoSmithKline (Marly-Le-Roi, France); rifampin, Aventis (Paris, France); trimethoprim and tetracycline, Sigma (Saint-Quentin Fallavier, France); kanamycin, Euromedex (Mundolsheim, France).

### Nucleic acid extractions

Circular DNA molecules were extracted using Plasmid Maxi Kits (Qiagen, Courtaboeuf, France) according to the instructions of the manufacturer. Extractions of whole-cell DNAs were done as described elsewhere [35].

### PCR experiments

*Taq* and *Pfu* DNA polymerases were from Roche Diagnostics (Meylan, France) and Promega Corporation (Madison, Wis.), respectively. PCR experiments [36] were performed using the series of primers listed in Table S1. The PCR products were purified using Qiaquick columns (Qiagen). To determine the insertion sites of the *veb1* cassette or derivatives into the In3 integron, plasmids from independent *E. coli* DH10B-Rif (R388::*veb1*) transconjugants were extracted and amplifications using combination of primers VEBINV2-TMPB, VEBINV2-ORFAB, VEBINV2-QACEB, and TMPA-VEBINV3 were performed. The *aadB* cassette was detected by PCR amplification using the AADBF and AADBB primers.

For each construct tested, five cointegrates resulting from cointegration between R388 and *veb1*-containing plasmids were analyzed. Amplifications using combination of primers T3-5′CS, T7 promoter-3′CS and VEBINV2-3′CS were performed in order to determine which recombination sites were involved in the formation of the cointegrates.

### Cloning experiments and electroporation

T4 DNA ligase, and restriction endonucleases were used according to the recommendations of the manufacturer (Amersham Biosciences, Orsay, France). The plasmid p112.Kan was constructed by inserting a HindIII-digested omega fragment (ΩKm) from plasmid pHP45Ω-Km [37], made of a kanamycin resistance gene (*aph*(*3*′)*-IIa*) flanked by transcriptional and translational termination sequences, into the HindIII site from the multiple cloning site of p112 plasmid (pTRC99A::*intI1*) [33]. The pTRC99A.Kan plasmid was constructed by removing the 1.2-kb EcoRI-BamHI fragment containing the *intI1* gene from p112.Kan, filling in its ends with *Pfu* DNA polymerase and followed by self ligation. The inserts of the recombinant plasmids pVeb and pVeb.aadB, corresponded to fragments of 1.6-kb (containing the *veb1* cassette) and 1.7-kb (containing the *veb1* and *aadB* cassettes) that were amplified with the pairs of primers VEBCASF/AADBB and VEBCASF/3′CS, respectively and genomic DNA of *P. aeruginosa* 14 as template (Figure S1). The inserts of the recombinant plasmids pAttI.veb and pAttI.veb.aadB corresponded to fragments of 2-kb (containing an entire *attI1* site and the *veb1* cassette) and 2.1-kb (containing an entire *attI1* site and the *veb1* and *aadB* cassettes) that were amplified with the pairs of primers INTIN/AADBB and INTIN/3′CS, respectively and genomic DNA of *P. aeruginosa* 14 as template (Figure S1). The inserts of the recombinant plasmids pAttI.IS.veb and pAttI.IS.veb.aadB corresponded to fragments of 3.4-kb (containing a disrupted *attI1* site, IS*1999*, and the *veb1* cassette) and 3.5-kb (containing a disrupted *attI1* site, IS*1999*, and the *veb1* and *aadB* cassettes) that were amplified with the pairs of primers INTIN/AADBB and INTIN/3′CS, respectively and genomic DNA of *P. aeruginosa* 15 as template (Figure S1). PCR products were purified prior to cloning into the SmaI-restricted pBBR1MCS.3 vector.

Three different modifications of the *veb1 attC* site (veb1 attC*, *veb1 attC* ^Δ^, and *veb1 attC'*) were generated. Creation of the veb1 attC* site was performed by using the attCVEB1 and attCVEB2 primers that anneal to the beginning of the *veb1 attC* site up to the inverse core site and to the beginning of the *aadB* gene cassette (core site), respectively. These primers have floating 5′ ends, each corresponding to a half of the *aadB attC* site, and have in common the portion containing the BsaHI restriction site (Figure S1). The recombinant plasmid pAttI.veb* containing veb1 attC* site was constructed as follows: a 1.5-kb fragment amplified with the pair of primers T7 promoter-attCVEB1 and pAttI.veb as template was digested with SpeI-BsaHI. A 0.7-kb fragment was amplified with the primers attCVEB2-T3 and pAttI.veb as template and was digested with BsaHI-PstI. Then, the digested inserts were purified and mixed in a three-way ligation with the PstI-SpeI-restricted pBBR1MCS.3 vector generating pAttI.veb* (Figure S1). The pAttI.veb.aadB and pAttI.veb* recombinant plasmids were digested with SacI endonucleases. The SacI insert of pAttI.veb* was cloned into the SacI-restricted pAttI.veb.aadB plasmid generating pAttI.veb*.aadB (Figure S1).

Similarly, pAttI.veb^Δ^ (containing *veb1 attC* ^Δ^) was constructed as follows: a 1.5-kb fragment amplified with the pair of primers T7 promoter-Shortattc1 and pAttI.veb as template was digested with SacI. A 0.6-kb fragment was amplified with the primers Shortattc2-T3 and pAttI.veb as template and was digested with KpnI. Then, the digested inserts were purified and mixed in a three-way ligation with the SacI-KpnI-restricted pBBR1MCS.3 vector generating pAttI.veb^Δ^ (Figure S1). The recombinant plasmid pAttI.veb' (containing *veb1 attC*') was constructed as follows: a 1.6-kb fragment amplified with the pair of primers T7 promoter-attc2L and pAttI.veb as template was digested with NdeI. The resulting 0.9-kb and 0.7-kb fragments were purified and subsequently digested with AvaI and SacI, respectively. A 0.3-kb fragment was amplified with the primers attc2R-T3 and pAttI.veb as template and was digested with AvaI-XhoI. Then, the digested inserts were purified and mixed before ligation with the SacI-XhoI-restricted pAttI.veb plasmid generating pAttI.veb' (Figure S1).

Ligation products were electroporated first into *E. coli* DH10B as previously described [35]. Selection was performed onto TS-agar plates containing tetracycline (15 µg/ml) and ceftazidime (15 µg/ml) except for pVeb and pVeb.aadB plasmids that were selected onto TS-agar plates containing tetracycline (15 µg/ml) only. Clones harboring recombinant plasmids pVeb, pVeb.aadB, pAttI.veb, pAttI.veb.aadB, pAttI.IS.veb, pAttI.IS.veb.aadB, pAttI.veb*, pAttI.veb*.aadB, pAttI.veb^Δ^, and pAttI.veb' were retained for further experiments (Figure S1).

### Sequencing

Sequencing was performed on both strands using laboratory-designed primers on an ABI PRISM 3100 automated sequencer (Applied Biosystems, Les Ullis, France).

### Induction of integrase expression

The recombinant plasmids pVeb, pVeb.aadB, pAttI.veb, pAttI.veb.aadB, pAttI.IS.veb, pAttI.IS.veb.aadB, pAttI.veb*, pAttI.veb*.aadB, pAttI.veb^Δ^, and pAttI.veb' were freshly electroporated into *E. coli* DH10B (p112.Kan); the plasmid pAttI.veb.aadB was also freshly electroporated into *E. coli* DH10B (pTRC99A.Kan) before each experiment. Strains were grown to stationary phase in TS-broth containing tetracycline 15 µg/ml and kanamycin 30 µg/ml. The cultures were then diluted 1000-fold into 200 ml TS-broth containing tetracycline 15 µg/ml, kanamycin 30 µg/ml and grown to exponential phase (OD 600 nm: 0.5). Integrase expression was then induced for 2 h by adding IPTG at a final concentration of 0.6 M.

### Detection of *veb1-*containing recombination products by hybridization

This assay allows for the detection of *veb-1* containing molecules that are the result of a recombination event between *attI1* (or disrupted *attI1*) and an *attC* site and gives an insight into their recombination efficiency. Total circular DNA content was extracted from *E. coli* DH10B (p112.Kan) strains harboring recombinant plasmids pVeb, pVeb.aadB, pAttI.veb, pAttI.veb.aadB, pAttI.IS.veb, pAttI.IS.veb.aadB, pAttI.veb*, pAttI.veb*.aadB, pAttI.veb^Δ^, and pAttI.veb' and *E. coli* DH10B (pTRC99A.Kan) harboring pAttI.veb.aadB after IPTG-induction. Five µg of circular DNA extracts were digested in duplicate with 10 units of BspEI, which cleaves at a unique site located in the *veb1* cassettes (Figure S1). Digested DNA samples were then loaded on two agarose gels (25 cm, 0.7%) and electrophoresed at 45 V for 16 h using Tris-borate-EDTA running buffer [36]. The duplicates that were loaded on the second gel were spaced two wells apart.

The first agarose gel was used for hybridization experiments as follows: The bottom section of the gel that contained small BspEI excision products (<2-kb), was cut and transferred onto a N^+^ Hybond nylon membrane (Amersham Biosciences). Southern blot hybridizations [36] were performed under high-stringency conditions using the ECL nonradioactive labeling and detection kit (Amersham Biosciences). The probe consisted of a PCR-generated fragment internal to *bla*_VEB-1_ that was amplified using primers VEB1A/VEB1B and whole-cell DNA of *P. aeruginosa* 14 as template.

The second agarose gel was used to extract BspEI digested *veb1*-containing fragments. Since DNA amounts were very low and could not be detected visually after ethidium bromide staining, DNA location was spotted by superposing the autoradiography film obtained from the first gel after hybridization. Gel slices were cut with a separate disposable scalpel to avoid sample cross-contamination. DNA was extracted using Qiaquick Gel extraction kit (Qiagen) and subjected to PCR amplification using the VEBINV3-VEBINV2 outward-directed primers. These primers are located on each side of the BspE1 restriction site, and thus allowed amplification of the recombinant junction that was created upon recombination (Figure S2 and S3).

### Cointegration assay using plasmid R388 and calculation of cointegration frequencies

Precise integration of *veb1* gene cassettes into the class 1 integron In3 carried by the conjugative plasmid R388 or cointegration between *veb1*-containing plasmids and R388 was investigated using a mating-out assay as follows. The recombinant plasmids pVeb, pVeb.aadB, pAttI.veb, pAttI.veb.aadB, pAttI.IS.veb, pAttI.IS.veb.aadB, pAttI.veb*, pAttI.veb*.aadB and pBBR1MCS.3 cloning vector were freshly electroporated into *E. coli* DH10B (p112.Kan; R388); the plasmid pAttI.veb.aadB was freshly electroporated into *E. coli* DH10B (pTRC99A.Kan; R388). Twenty-four hours after electroporation, three single colonies (for each plasmid tested) were independently cultured overnight at 37°C in 10 ml TS broth containing tetracycline, 15 µg/ml and kanamycin, 30 µg/ml. The overnight cultures were diluted 10-fold in fresh TS broth without antibiotic and were cultured under low agitation at 37°C for 1 h 30, prior to a 2 h IPTG-induction (induction of *intI1* expression). Mating was performed by incubating 800 µl of recipient *E. coli* DH10B-Rif and 200 µl of the tested strain under low agitation at 37°C for 3 h. Subsequently, the mating mixture was vigorously vortexed, placed on ice, and plated. One hundred microliters aliquots of serial ten fold dilutions were plated onto trimethoprim- (25 µg/ml) and rifampin- (200 µg/ml) containing plates and ceftazidime- (15 µg/ml) and rifampin- (200 µg/ml) containing plates. For *E. coli* DH10B (R388; p112.Kan; pBBR1MCS.3) aliquots were plated onto trimethoprim- (25 µg/ml) and rifampin- (200 µg/ml) containing plates and tetracycline- (15 µg/ml) and rifampin- (200 µg/ml) containing plates. The cointegration frequency was calculated by dividing the number of ceftazidime and rifampin resistant (CazR-RifR) transconjugants by the number of trimethoprim and rifampin resistant (TmpR-RifR) transconjugants. For each plasmid tested, up to 484 CazR-RifR colonies were further screened for tetracycline susceptibility in order to discriminate cointegrates from precise *veb1* cassettes integration into In3.

For *E. coli* DH10B (R388; p112.Kan; pBBR1MCS.3) the cointegration frequency was calculated by dividing the number of tetracycline and rifampin resistant (TetR-RifR) transconjugants by the number of TmpR-RifR transconjugants.

## Supporting Information

Figure S1 **Schematic representation of the plasmid constructs used in this study.** All constructs were cloned into the multiple cloning site of the pBBR1MCS.3 shuttle-vector represented with a solid line. The coding regions are shown as boxes with an arrow indicating the orientation of their transcription. Dashed lines indicate truncated genes. The black diamond, white, grey, white with black dots, and black with white dots circles represent *attI1*, *veb1 attC, aadB attC, veb1 attC* ^Δ^, and *veb1 attC*', respectively. The veb1 attC* site is highly similar to the *aadB attC* site and is also represented by a grey circle. The IS*1999* inverted repeats are shown by empty triangles. The broken arrows indicate the P*_c_*, P*_out_* and P*_lac_* promoters. Restriction sites used for cloning are indicated on the pAttI.veb.aadB representation. Small arrows (1 and 2) located on each side of the BspEI restriction site represent the positions of the VEBINV3 and VEBINV2 primers, respectively.
(TIF)

Figure S2 **Recombination products obtained with pAttI.veb.** A) Schematic representation of plasmid pAttI.veb. Construct was made in the pBBR1MCS.3 shuttle-vector represented with a solid line. The coding regions are shown as boxes with an arrow indicating the orientation of their transcription. Dashed lines indicate truncated genes. The black diamond and white circle represent *attI1* and *veb1 attC*, respectively. The broken arrow indicates the P*_c_* promoter. Small arrows (1 and 2) located on each side of the BspEI restriction site represent the positions of the VEBINV3 and VEBINV2 primers, respectively. B–D) Recombination products. Each possible recombination product is shown. The sites involved in the recombination and the size of the relevant BspEI digestion products are indicated. B) *Veb1* cassette excision product. C) Cointegrates. Double lines represent scale breaks. D) Gene duplications. *Veb1* duplication can arise from integration (*attI* x *veb1 attC* or *veb1 attC* x *veb1 attC*) of an excised *veb1* gene cassette, or by resolution (*veb1 attC* x *attI1* or *attI1* x *attI1*) of cointegrates. In any case, the 1.1-kb fragment is only recovered after BspEI digestion when recombination between *attI1* and *veb1 attC* has occurred.
(TIF)

Figure S3 **Recombination products obtained with pAttI.veb.aadB.** A) Schematic representation of plasmid pAttI.veb.aadB. Construct was made in the pBBR1MCS.3 shuttle-vector represented with a solid line. The coding regions are shown as boxes with an arrow indicating the orientation of their transcription. Dashed lines indicate truncated genes. The black diamond, white and grey circles represent *attI1, veb1 attC* and *aadB attC*, respectively. The broken arrow indicates the P*_c_* promoter. Small arrows (1 and 2) located on each side of the BspEI restriction site represent the positions of the VEBINV3 and VEBINV2 primers, respectively. B–D) Recombination products. The different *veb1*-containing recombination products are represented. The sites involved in the recombination and the size of the relevant BspEI digestion products are indicated. B) *Veb1*-containing excision product. C) Cointegrates. Double lines represent scale breaks. D) Gene duplications. *Veb1* duplications can arise from integration of an excised *veb1*-containing gene cassette or by resolution of cointegrates. In any case, the 1.1-kb and 1.6-kb fragments are only recovered after BspEI digestion when recombinations *attI1* x *veb1 attC* and *attI1* x *aadB attC* have occurred, respectively.
(TIF)

Table S1 **Sequence of primers used in this study.** ^a^ Nucleotides that are complementary to the *aadB attC* site are underlined; ^b^ Restriction sites are bolded; ^c^ Nucleotides that are mutated are boxed in grey. Supplemental references are as follows: (1). Naas T, Coignard B, Carbonne A, Blanckaert K, Bajolet O, et al. (2006) VEB-1 Extended-spectrum beta-lactamase-producing *Acinetobacter baumannii*, France. Emerg Infect Dis 12: 1214–1222. (2). Girlich D, Naas T, Leelaporn A, Poirel L, Fennewald M, et al. (2002) Nosocomial spread of the integron-located *veb-1*-like cassette encoding an extended-pectrum beta-lactamase in *Pseudomonas aeruginosa* in Thailand. Clin Infect Dis 34: 603–611. (3). Martinez E, de la Cruz F (1990) Genetic elements involved in Tn*21* site-specific integration, a novel mechanism for the dissemination of antibiotic resistance genes. EMBO J 9: 1275–1281.
(DOCX)
