# Supplementary figures and images for: Integrase-Mediated Recombination of the veb1 Gene Cassette Encoding an Extended-Spectrum β-Lactamase
Source: PLoS One. 2012 Dec 10;7(12):e51602. doi: 10.1371/journal.pone.0051602 (PMC3518468; doi:10.1371/journal.pone.0051602)

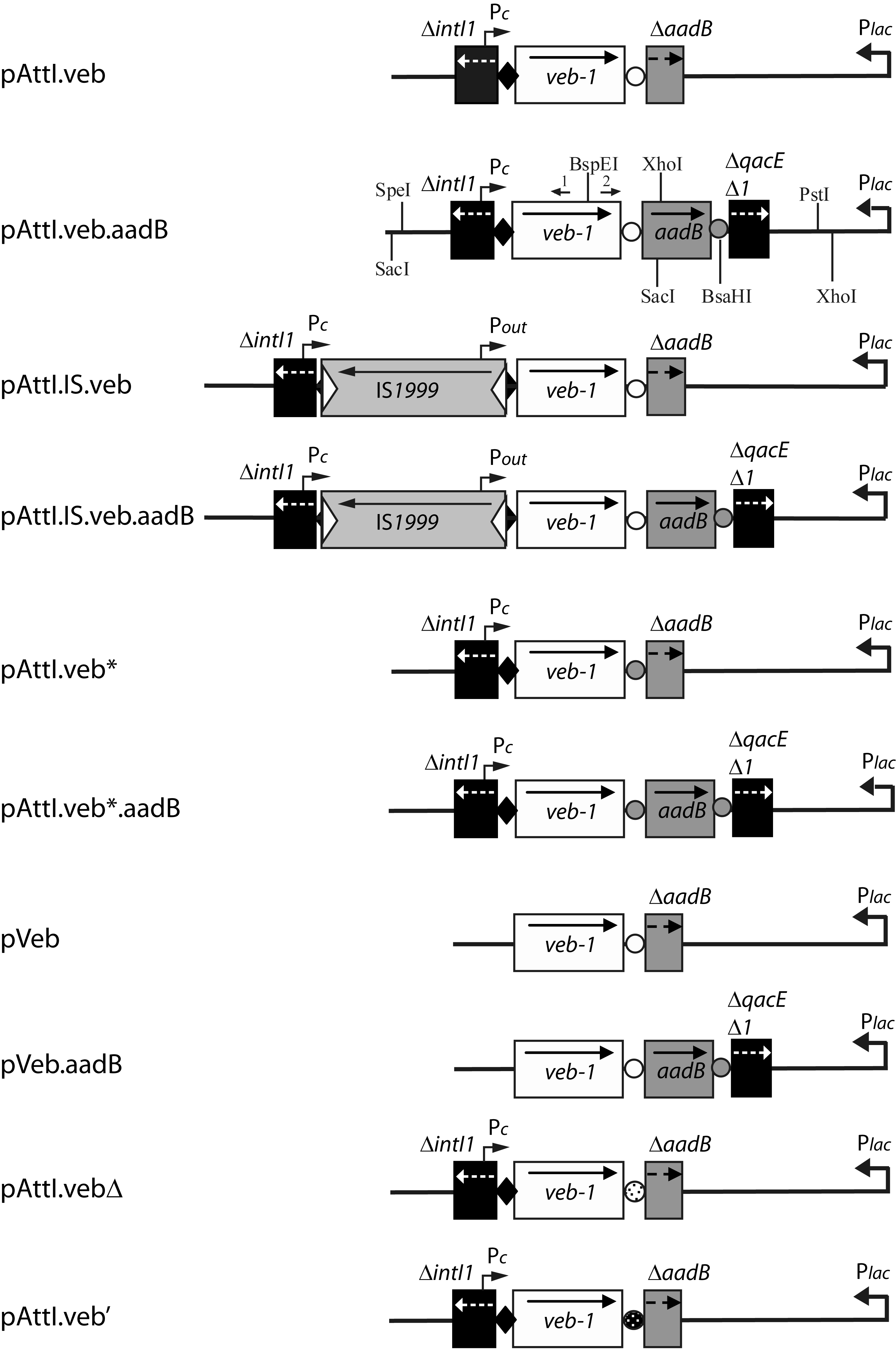

Supplement: Figure S1 — Schematic representation of the plasmid constructs used in this study. All constructs were cloned into the multiple cloning site of the pBBR1MCS.3 shuttle-vector represented with a solid line. The coding regions are shown as boxes with an arrow indicating the orientation of their transcription. Dashed lines indicate truncated genes. The black diamond, white, grey, white with black dots, and black with white dots circles represent attI1, veb1 attC, aadB attC, veb1 attC Δ, and veb1 attC', respectively. The veb1 attC* site is highly similar to the aadB attC site and is also represented by a grey circle. The IS1999 inverted repeats are shown by empty triangles. The broken arrows indicate the Pc, Pout and Plac promoters. Restriction sites used for cloning are indicated on the pAttI.veb.aadB representation. Small arrows (1 and 2) located on each side of the BspEI restriction site represent the positions of the VEBINV3 and VEBINV2 primers, respectively. (TIF) [file pone.0051602.s001.tif]

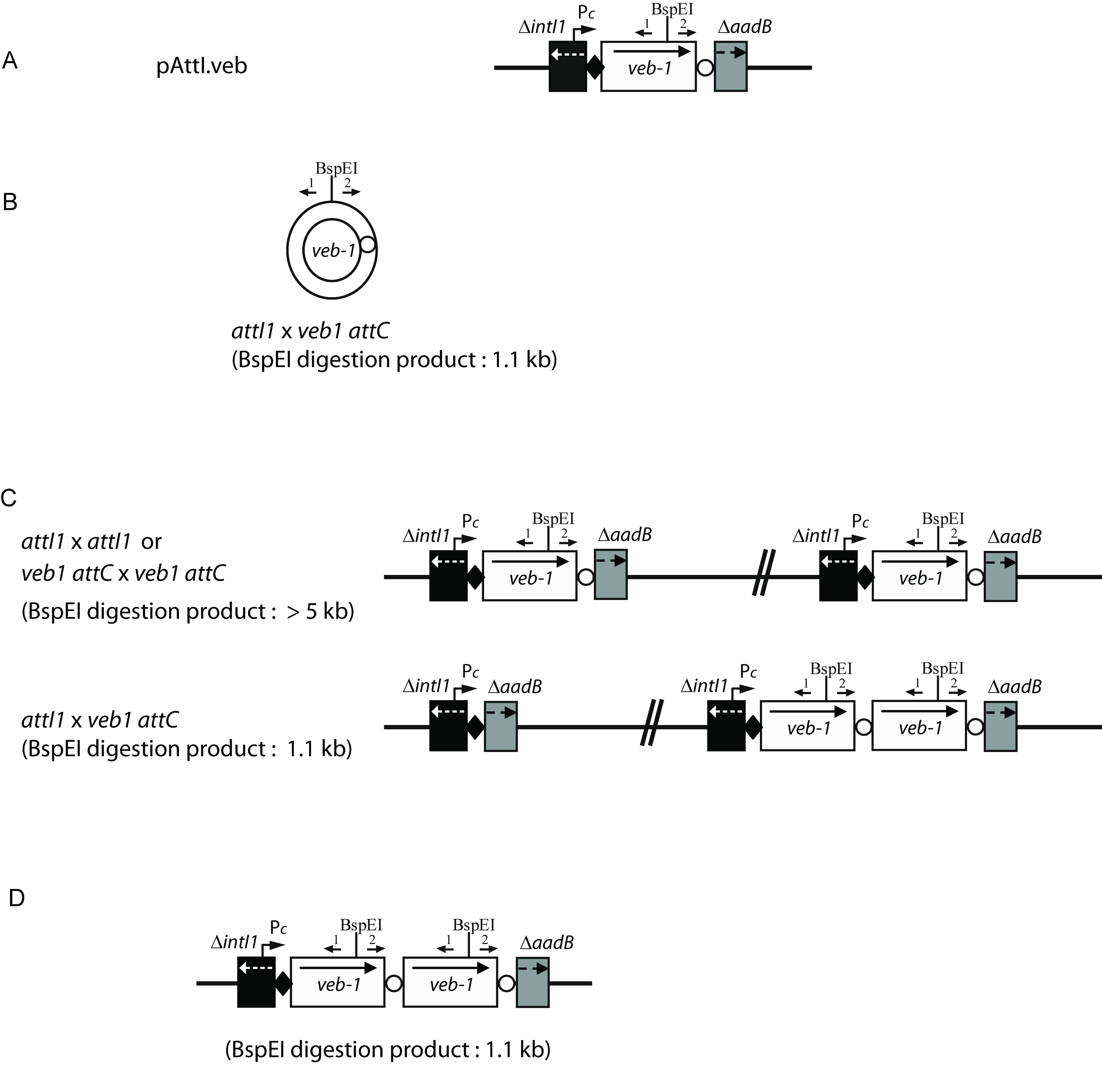

Supplement: Figure S2 — Recombination products obtained with pAttI.veb. A) Schematic representation of plasmid pAttI.veb. Construct was made in the pBBR1MCS.3 shuttle-vector represented with a solid line. The coding regions are shown as boxes with an arrow indicating the orientation of their transcription. Dashed lines indicate truncated genes. The black diamond and white circle represent attI1 and veb1 attC, respectively. The broken arrow indicates the Pc promoter. Small arrows (1 and 2) located on each side of the BspEI restriction site represent the positions of the VEBINV3 and VEBINV2 primers, respectively. B–D) Recombination products. Each possible recombination product is shown. The sites involved in the recombination and the size of the relevant BspEI digestion products are indicated. B) Veb1 cassette excision product. C) Cointegrates. Double lines represent scale breaks. D) Gene duplications. Veb1 duplication can arise from integration (attI x veb1 attC or veb1 attC x veb1 attC) of an excised veb1 gene cassette, or by resolution (veb1 attC x attI1 or attI1 x attI1) of cointegrates. In any case, the 1.1-kb fragment is only recovered after BspEI digestion when recombination between attI1 and veb1 attC has occurred. (TIF) [file pone.0051602.s002.tif]

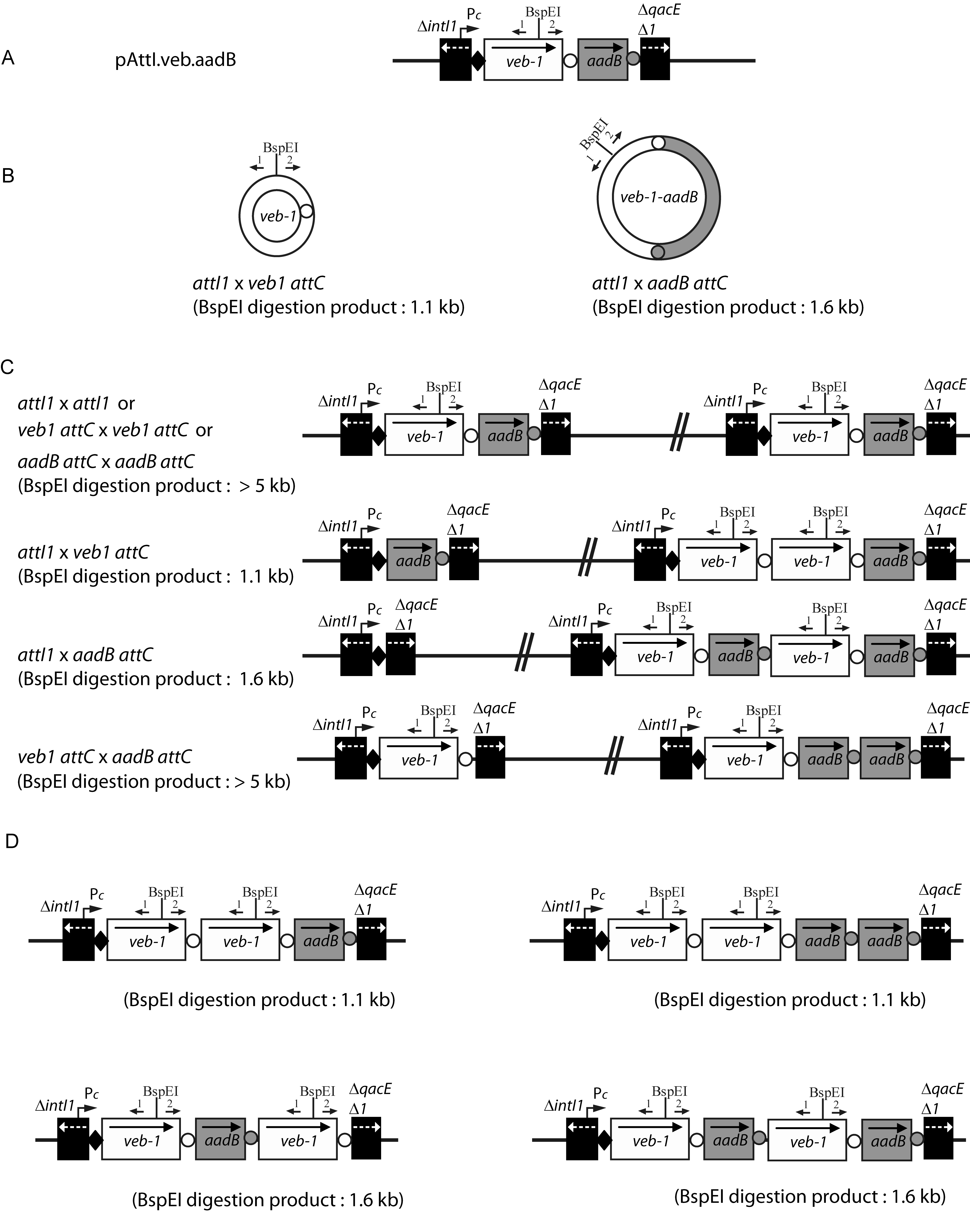

Supplement: Figure S3 — Recombination products obtained with pAttI.veb.aadB. A) Schematic representation of plasmid pAttI.veb.aadB. Construct was made in the pBBR1MCS.3 shuttle-vector represented with a solid line. The coding regions are shown as boxes with an arrow indicating the orientation of their transcription. Dashed lines indicate truncated genes. The black diamond, white and grey circles represent attI1, veb1 attC and aadB attC, respectively. The broken arrow indicates the Pc promoter. Small arrows (1 and 2) located on each side of the BspEI restriction site represent the positions of the VEBINV3 and VEBINV2 primers, respectively. B–D) Recombination products. The different veb1-containing recombination products are represented. The sites involved in the recombination and the size of the relevant BspEI digestion products are indicated. B) Veb1-containing excision product. C) Cointegrates. Double lines represent scale breaks. D) Gene duplications. Veb1 duplications can arise from integration of an excised veb1-containing gene cassette or by resolution of cointegrates. In any case, the 1.1-kb and 1.6-kb fragments are only recovered after BspEI digestion when recombinations attI1 x veb1 attC and attI1 x aadB attC have occurred, respectively. (TIF) [file pone.0051602.s003.tif]
