# Supplementary material for: Integrase-Mediated Recombination of the veb1 Gene Cassette Encoding an Extended-Spectrum β-Lactamase
Source: PLoS One. 2012 Dec 10;7(12):e51602. doi: 10.1371/journal.pone.0051602 (PMC3518468; doi:10.1371/journal.pone.0051602)
Supplement: Table S1 — Sequence of primers used in this study. a Nucleotides that are complementary to the aadB attC site are underlined; b Restriction sites are bolded; c Nucleotides that are mutated are boxed in grey. Supplemental references are as follows: (1). Naas T, Coignard B, Carbonne A, Blanckaert K, Bajolet O, et al. (2006) VEB-1 Extended-spectrum beta-lactamase-producing Acinetobacter baumannii, France. Emerg Infect Dis 12: 1214–1222. (2). Girlich D, Naas T, Leelaporn A, Poirel L, Fennewald M, et al. (2002) Nosocomial spread of the integron-located veb-1-like cassette encoding an extended-pectrum beta-lactamase in Pseudomonas aeruginosa in Thailand. Clin Infect Dis 34: 603–611. (3). Martinez E, de la Cruz F (1990) Genetic elements involved in Tn21 site-specific integration, a novel mechanism for the dissemination of antibiotic resistance genes. EMBO J 9: 1275–1281. (DOCX) [file pone.0051602.s004.docx]

**TABLE S1. Sequence of primers used in this study**

| **Primer** | **Sequence (5’→3’)** ^a, b, c^ | **Accession number** | **Primer position** | **Reference** |
| --- | --- | --- | --- | --- |
| T3 | ATTAACCCTCACTAAAGGGA | U25059 | 3352-3335 | Universal primer |
| T7 promoter | AATACGACTCACTATAGG | U25059 | 3189-3206 | Universal primer |
| INTIN | GCCAGGGCAGATCCGTGCAC | AF133699 | 716-735 | This work |
| VEB1A | CGACTTCCATTTCCCGATGC | AF133699 | 3895-3914 | [[1](#_ENREF_1)] |
| VEB1B | GGACTCTGCAACAAATACGC | AF133699 | 4537-4518 | [[1](#_ENREF_1)] |
| VEBCASF | gttagcggtaatttaaccagatag | AF133699 | 3680-3703 | [[2](#_ENREF_2)] |
| VEBINV2 | agcgtatttgttgcagagtcc | AF133699 | 4517-4537 | [[2](#_ENREF_2)] |
| VEBINV3 | gaacagaatcagttcctccg | AF133699 | 4163-4144 | [[2](#_ENREF_2)] |
| attCVEB1 | GTTAAGCCGCGCCGCGAAGC**GGCGTC**GGCTTGGACGAATTGTTAGCGGTAGTTTTTTATTTATTCAAATAGTAATTCCACG | AF133699 | 4630-4590 | This work |
| attCVEB2 | CCAAGCC**GACGCC**GCTTCGCGGCGCGGCTTAACTCAGGTGTTAGGCCGCATGGACACAACGCAGGTCACATTGATACAC | AF133699 | 4750-4789 | This work |
| ShortattC1 | CCCGCCATTGCCTATGAGCCAGTGTTAGCGGTAGTT | AF133699 | 4651-4618 | This work |
| ShortattC2 | cgccactgcccatagcccaaaccgttaggccgcatg | AF133699 | 4727-4762 | This work |
| attC2L | CG**CTCGGG**CGGGCTACCGACTTTGCAAATTGCACTTCAACCCGCCATTGGCTTGGACCCAGTGTTAGCGGTAGTT | AF133699 | 4692-4618 | This work |
| attc2R | g**cccgag**cgttttctcggtttgacaggaaaggctcacgcaaaccgccactgccGCtTAAccaaaccgttaggccgcatg | AF133699 | 4684-4762 | This work |
| AADBF | gatacacaaaattctagctgcg | AF133699 | 4783-4804 | [[3](#_ENREF_3)] |
| AADBB | cgcatatcgcgacctgaaagc | AF133699 | 5287-5267 | [[3](#_ENREF_3)] |
| 5’CS | GGCATCCAAGCAGCAAG | AF133699 | 1056-1072 | [[2](#_ENREF_2)] |
| 3’CS | AAGCAGACTTGACCTGA | AF133699 | 5397-5381 | [[2](#_ENREF_2)] |
| TMPA | atgggtcaaagtagcgatgaagcc | U12441 | 3089-3112 | This work |
| TMPB | ttaggccacacgttcaagtgc | U12441 | 3325-3305 | This work |
| ORFAB | TTAGGCGTCATTCTTCCAACCCCGA | U12441 | 3814-3790 | This work |
| QACEB | GTTCGAAAACGGGTACTTCG | U12441 | 4229-4210 | [[2](#_ENREF_2)] |
